# Phosphoproteome analysis reveals the involvement of protein dephosphorylation in ethylene-induced corolla senescence in petunia

**DOI:** 10.1186/s12870-021-03286-x

**Published:** 2021-11-03

**Authors:** Shiwei Zhong, Lina Sang, Zhixia Zhao, Ying Deng, Haitao Liu, Yixun Yu, Juanxu Liu

**Affiliations:** 1 Guangdong Key Laboratory for Innovative Development and Utilization of Forest Plant Germplasm, College of Forestry and Landscape Architecture, South China Agricultural University, Guangzhou, 510642 China; 2 School of Landscape Architecture School of Tourism and Health, Zhejiang A & F University, Zhejiang, 311300 Hangzhou China; 3 Lingnan Guangdong Laboratory of Modern Agriculture, Guangzhou, 510642 China

**Keywords:** Ethylene, Phosphorylation, Senescence, Petunia, Dephosphorylation, Alternative splicing

## Abstract

**Background:**

Senescence represents the last stage of flower development. Phosphorylation is the key posttranslational modification that regulates protein functions, and kinases may be more required than phosphatases during plant growth and development. However, little is known about global phosphorylation changes during flower senescence.

**Results:**

In this work, we quantitatively investigated the petunia phosphoproteome following ethylene or air treatment. In total, 2170 phosphosites in 1184 protein groups were identified, among which 2059 sites in 1124 proteins were quantified. To our surprise, treatment with ethylene resulted in 697 downregulated and only 117 upregulated phosphosites using a 1.5-fold threshold (FDR < 0.05), which showed that ethylene negatively regulates global phosphorylation levels and that phosphorylation of many proteins was not necessary during flower senescence. Phosphoproteome analysis showed that ethylene regulates ethylene and ABA signalling transduction pathways via phosphorylation levels. One of the major targets of ethylene-induced dephosphorylation is the plant mRNA splicing machinery, and ethylene treatment increases the number of alternative splicing events of precursor RNAs in petunia corollas.

**Conclusions:**

Protein dephosphorylation could play an important role in ethylene-induced senescence, and ethylene treatment increased the number of AS precursor RNAs in petunia corollas.

**Supplementary Information:**

The online version contains supplementary material available at 10.1186/s12870-021-03286-x.

## Background

Senescence represents the last stage of flower development [1, 2] and is regulated by the levels of mRNA, mRNA modification, proteins and posttranslational modifications (PTMs) [3–5]. Ethylene is a gaseous plant hormone that significantly affects flower senescence [6]. Ethylene treatment changed the transcriptome, proteome, and metabolome profiles in higher plants [7–11]. Cytokinin 6-benzylaminopurine (BA) treatment prolonged petunia (*Petunia hybrida*) flower life and changed the transcript profile of flowers [12]. Differentially expressed transcripts and proteins between corollas the of pollinated petunia flowers and their unpollinated controls were identified by RNA sequencing and two-dimensional gel electrophoresis (2-DE), respectively [13, 14].

Phosphorylation is a highly dynamic and reversible PTM that plays an important role in plant growth and development, and it is involved in many signalling events and controlled by the activity of a large number of kinases (phosphorylation) and phosphatases (dephosphorylation) [15]. Generally, more kinases than phosphatases may be required since protein kinases play a key role in the response to a number of stimuli, while the function of phosphatases is only to reverse the effect of kinases [16]. During ageing of Arabidopsis, the expression of numerous protein kinases and phosphatases is altered and protein phosphorylation can regulate plant senescence [17]. However, our understanding of plant phosphoproteomes during flower senescence remains very limited with respect to their complexity and functions.

Several key kinases showed a key role in modulating leaf senescence. ENHANCED DISEASE RESISTANCE1 protein kinase negatively regulates ethylene-induced senescence in an ETHYLENE INSENSITIVE2 (EIN2)-dependent manner [18, 19]. ARABIDOPSIS HISTIDINE KINASE3 is involved in cytokinin-mediated control of leaf longevity through the specific phosphorylation of ARABIDOPSIS RESPONSE REGULATOR2 (Kim et al., 2006). The Arabidopsis mitogen-activated protein kinase cascade MKK9-MPK6 plays a positive role in regulating leaf senescence [20]. Arabidopsis mutation of RPK1, which encodes the membrane-bound receptor protein kinase, delays age-dependent and ABA-induced senescence [21]. *Glycine max* SENESCENCE-ASSOCIATED RECEPTOR-LIKE KINASE (GmSARK) and its Arabidopsis homologue AtSARK regulate leaf senescence through synergistic actions of ethylene and auxin [22]. The ACTIVITY OF BC1 COMPLEX (ABC1) protein kinase OsABC1–2 is involved in dark-induced senescence in rice (*Oryza sativa*) [23].

Dephosphorylation by protein phosphatases functions as a balancing switch to reverse the effects of phosphorylation by protein kinases. Protein phosphatases also play an important role in the regulation of leaf senescence. In Arabidopsis, the PP2C family protein phosphatase SENESCENCE-ASSOCIATED GENE113 (SAG113) is involved in the control of water loss during leaf senescence [24]. The protein phosphatase MITOGEN-ACTIVATED PROTEIN KINASE PHOSPHATASE2 (MKP2) positively regulates oxidative stress tolerance, inactivates the MPK3 and MPK6 mitogen-activated protein kinases in Arabidopsis, and promotes early senescence [25, 26].

Petunia has become a model plant for flower senescence research [27] because it is a climacteric flower that undergoes a burst in respiration and ethylene production in association with flower senescence. We previously characterized the ubiquitination of ethylene-mediated senescence in petunia corollas [28].

**Fig. 1:**
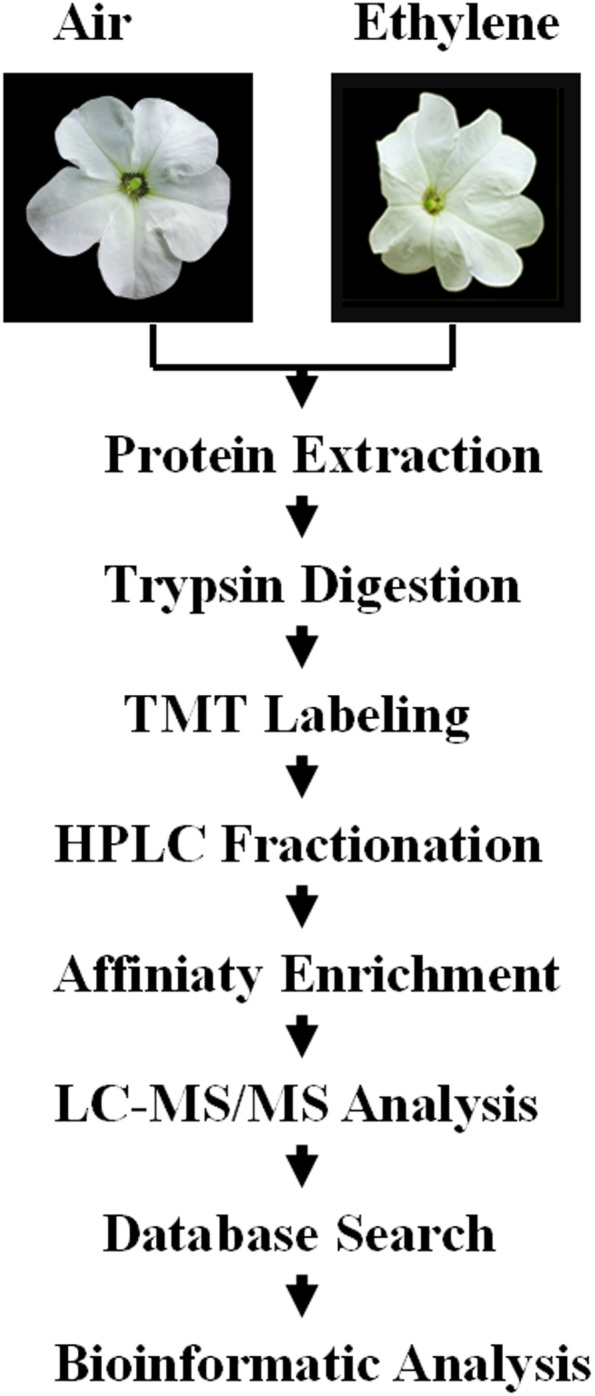
Systematic workflow for quantitative profiling of the global phosphoproteome in petunia corollas upon ethylene treatment

Here, parallel to the proteome and ubiquitylome response to ethylene treatment in petunia corollas previously reported by our group [28], an iTRAQ strategy involving antibody-based affinity enrichment and high-resolution LC-MS/MS analysis was used to perform a phosphoproteome analysis of petunia corollas with ethylene and air treatments (Fig. 1). In total, 1443 phosphosites were quantified as changing in response to 16 h of ethylene treatment in petunia corollas. Ethylene treatment greatly decreased the global phosphorylation levels in petunia corollas, implying that dephosphorylation of many proteins occurs and is probably necessary during flower senescence. The effects of ethylene treatment on the phosphorylation of proteins involved in hormone signal transduction, RNA metabolism and other processes were also discussed.

## Results

### Ethylene treatment changes the proteome profile in petunia corollas

To normalize the phosphorylome of petunia corollas treated with air or 2 μl l^− 1^ ethylene for 16 h, the tandem mass spectra of proteome data of petunia corollas were searched against the petunia genome (https://solgenomics.net/organism/Petunia_axillaris/genome), and these tandem mass spectra were also previously searched against these sequences of the transcriptome (SRA accession: SRP077541) that was previously constructed by our group to analyse the petunia proteome [28]. In total, 5663 protein groups were identified from petunia corollas, among which 4509 proteins were quantified. A total of 234 proteins were downregulated and 200 proteins were upregulated (with a threshold of 1.5-fold) in response to ethylene (*P* < 0.05), and a high degree of repeatability was observed (Supplementary data Table 1) [28].

### Ethylene treatment greatly decreases global phosphorylation levels in petunia corollas

**Fig. 2:**
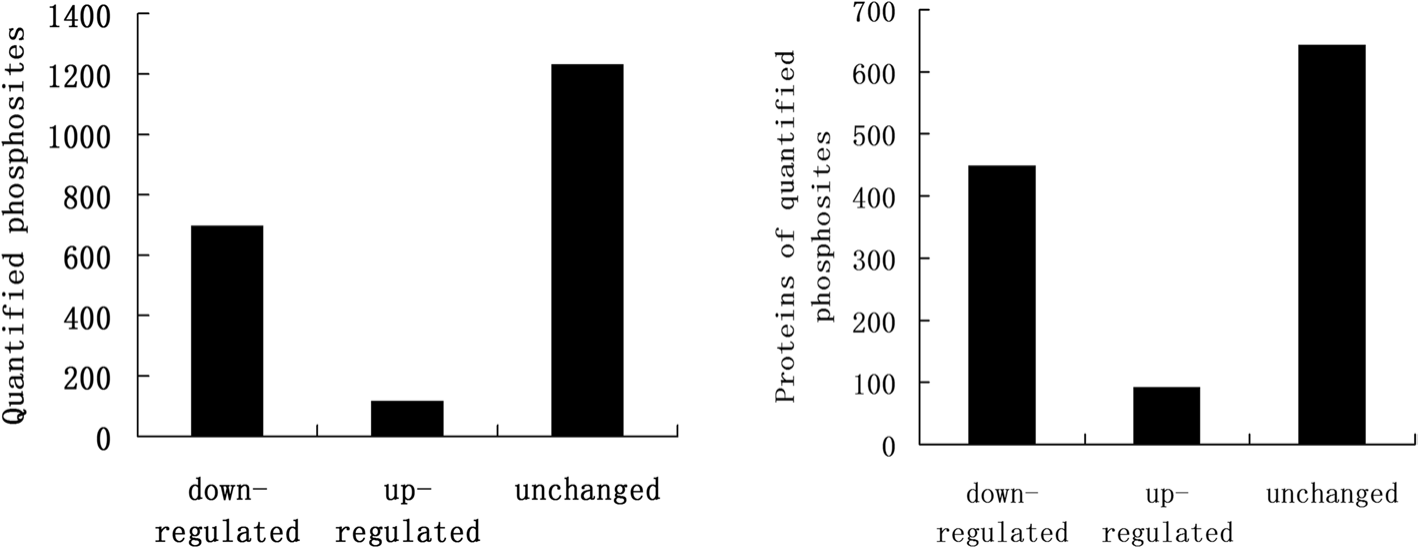
Ethylene treatment greatly decreases global phosphorylation levels in petunia corollas. The corollas were treated with air or 2 μl l^− 1^ ethylene for 16 h and then used for phosphorylation analysis with a threshold of 1.5-fold (*P* < 0.05)

The global phosphorylation response to ethylene was investigated in the corollas of petunias. Tandem mass spectra were searched against the petunia genome (https://solgenomics.net/organism/Petunia_axillaris/genome). The corollas were treated with air or 2 μl l^− 1^ ethylene for 16 h and used for the phosphorylation analysis. To increase the accuracy of phosphorylation, three biological replicates were analysed for each sample. Altogether, 2170 phosphosites in 1184 protein groups were identified (Supplementary data Table 2), among which 2159 sites in 1124 proteins were quantified after normalization based on the proteome data. To our surprise, 697 phosphosites in 449 proteins were downregulated, and only 117 phosphosites in 92 proteins were upregulated, with a threshold of 1.5-fold (FDR < 0.05) and high repeatability (Fig. 2, Supplementary Fig. S1, Supplementary data Table 3), suggesting that ethylene negatively regulates phosphorylation in petunia corollas. Of the 2170 nonredundant phosphosites, 84.9% (1843) were phosphorylated at serine residues, 14.1% (307) at threonine residues, and 0.9% (20) at tyrosine residues. This finding is consistent with previous reports in Arabidopsis and soybean [29, 30].

Several spectra corresponding to sites from proteins that undergo phosphorylation are presented in Supplementary Fig. S2.

### GO, KEGG pathway, and protein domain and subcellular localization analyses of phosphoproteins

**Fig. 3:**
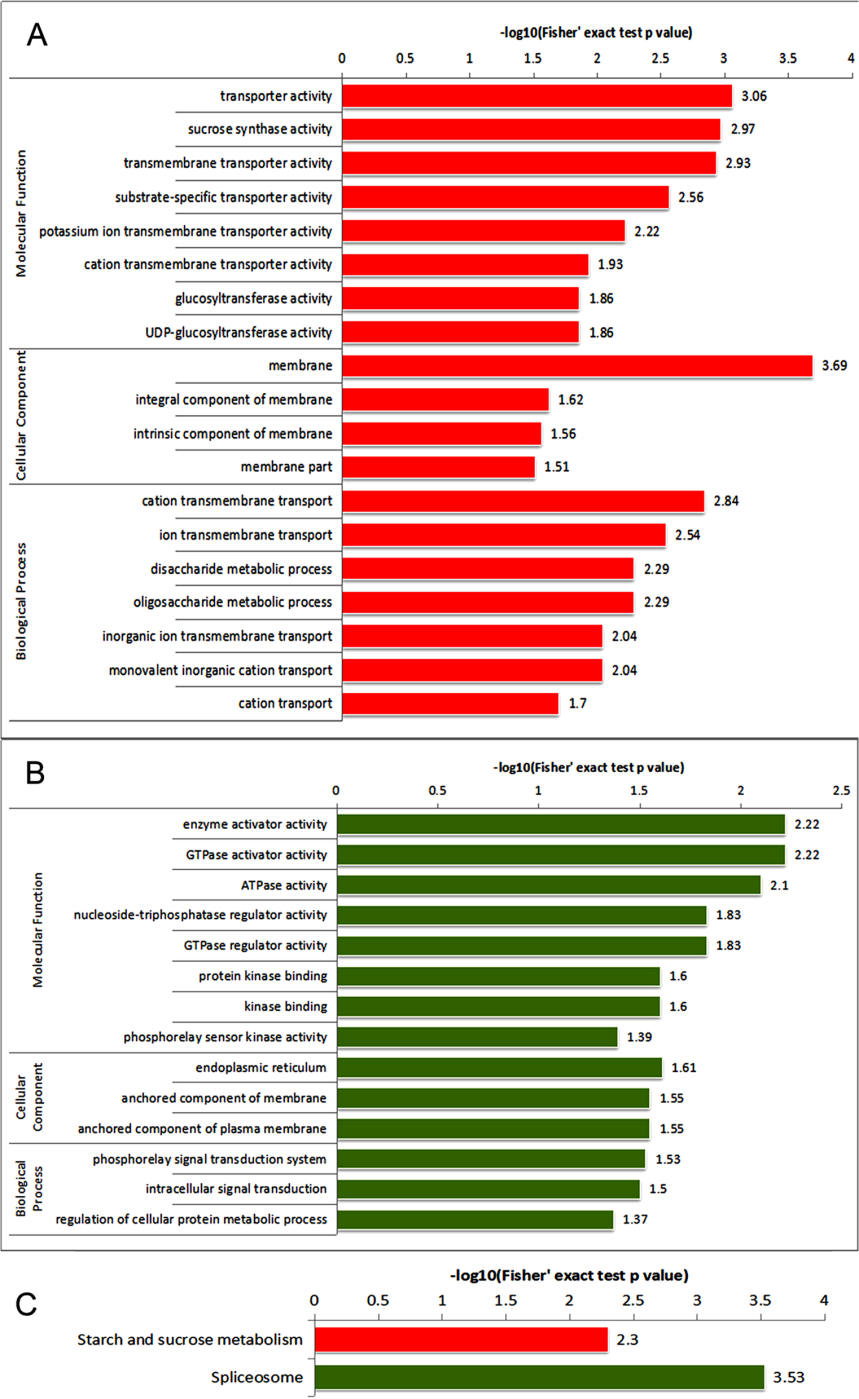
Functional enrichment analysis of proteins with differentially expressed phosphosites. A and B, GO-based enrichment analysis of proteins with downregulated (A) and upregulated (B) phosphosites. C, KEGG pathway-based enrichment analysis of proteins with downregulated phosphosites. Abbreviations: BP, Biological Process; MF, Molecular Function; and CC, Cellular Component

**Fig. 4:**
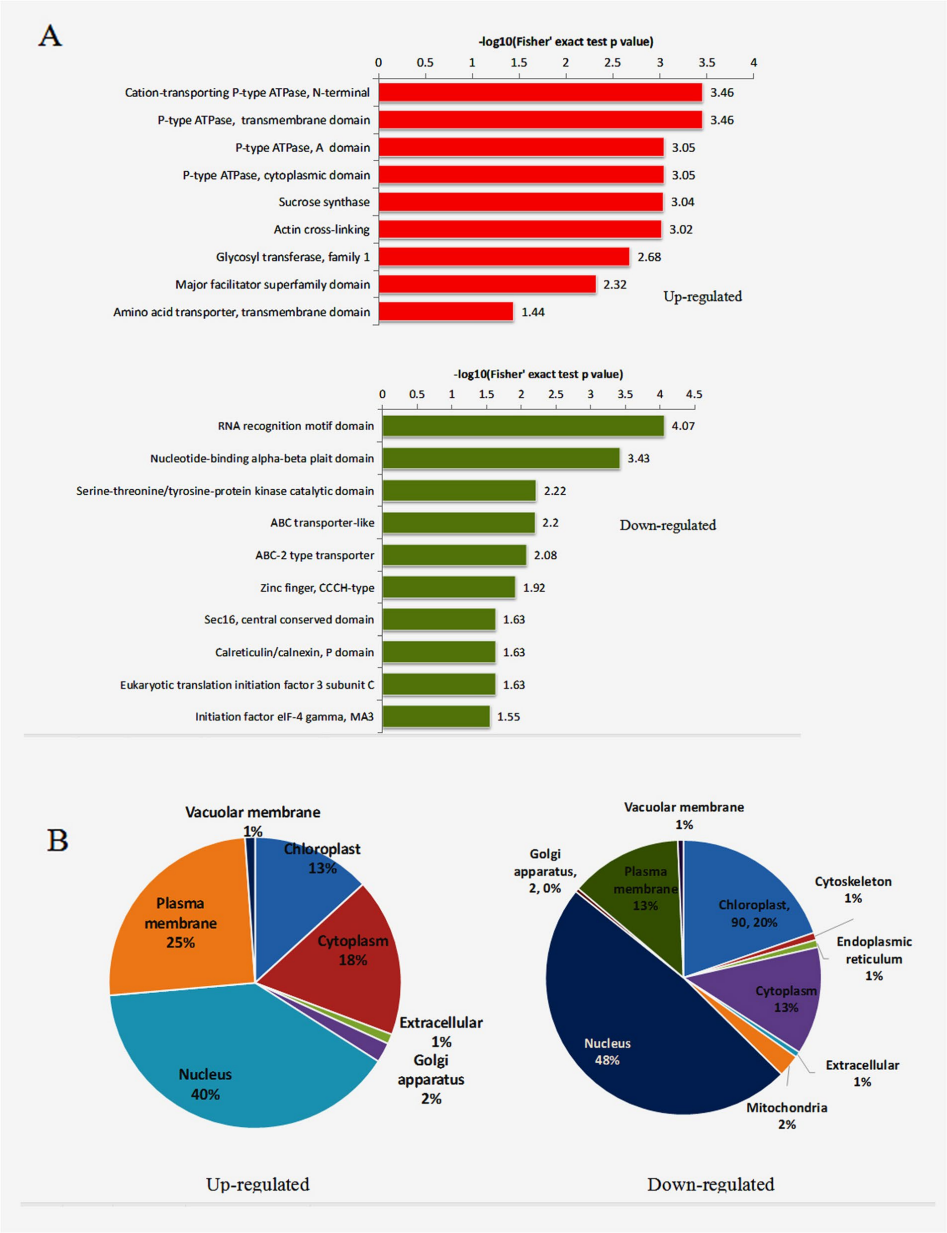
Protein domain (**A)** and subcellular location (**B**) enrichment analyses of proteins with up- (left) and downregulated (right) phosphorylation sites

To identify the functions of these phosphoproteins, we performed Gene Ontology **(**GO), Kyoto Encyclopedia of Genes and Genomes (KEGG) pathway, and protein domain and subcellular localization analyses (Fig. 3-4; Supplementary data Table 4, 5, 6).

A total of 116 proteins (389 different phosphosites) from corollas treated with and without ethylene were assigned GO annotations. In the cellular component category (Fig. 3A and B, Supplementary data Table 4), both the upregulated and downregulated proteins were highly enriched in the membrane, such as integral components of the membrane and intrinsic components of the membrane and membrane, implying that phosphorylation in response to ethylene may play important roles in cell membranes.

In the biological process analysis, several of the downregulated phosphoproteins were highly enriched in protein modification processes, translational initiation and phosphorus metabolic process regulation. Proteins with upregulated phosphosites were highly enriched in transmembrane transport and sucrose metabolic processes (Fig. 3A and B, Supplementary data Table 4), indicating the important role of cation transmembrane transport during ethylene-induced senescence.

The molecular function analysis shows that proteins with catalytic activity and binding were enriched in proteins with downregulated phosphosites, which is consistent with the impact of ethylene treatment on protein modification and transcription regulation described above (Fig. 3A and B, Supplementary data Table 3). Proteins with transporter activity had upregulated phosphosites, suggesting an increase in the activity of transporters following ethylene treatment, which is consistent with the increased activity of transporters in ethylene-mediated petal senescence [3].

The KEGG pathway analysis of the quantitatively changed proteins undergoing phosphorylation showed that the spliceosome pathway was enriched in proteins with downregulated sites. The starch and sucrose metabolism pathway was enriched in proteins with upregulated sites (Fig. 3C, Supplementary data Table 5). These results showed that phosphorylation was highly associated with RNA processing and sucrose metabolism.

For the protein domain analysis, we observed that proteins containing an RNA recognition motif domain, nucleotide-binding alpha-beta plait domain, ABC transporter-like, ABC-2 type transporter, Sec16, central conserved domain, calreticulin/calnexin, initiation factor eIF-4 gamma, zinc finger, and histidine kinase domain were enriched in proteins with downregulated phosphosites. Proteins containing cation-transporting P-type ATPase, P-type ATPase, transmembrane domain, P-type ATPase, sucrose synthase, actin cross-linking, glycosyl transferase of family 1, and transmembrane domain of amino acid transporter were enriched in upregulated quantiles (Fig. 4A, Supplementary data Table 6). These results suggested that ethylene treatment changed RNA recognition, transcription, kinase activity and transmembrane transport.

Subcellular localization analysis of the identified proteins indicated that most of the phosphoproteins with different phosphosites were nuclear (Fig. 4B), suggesting that phosphorylation may play a regulatory role in transcription initiation and elongation, RNA splicing, and DNA repair. In addition, many of the identified proteins were localized to the chloroplast, cytosol and plasma membrane.

### Sequence properties of the phosphoproteins

To understand the properties of the phosphosites identified in petunia, the Motif-X program was used to compare the position-specific frequencies of the amino acid residues surrounding all phosphorylated Ser/Thr/Tyr residues.

**Fig. 5:**
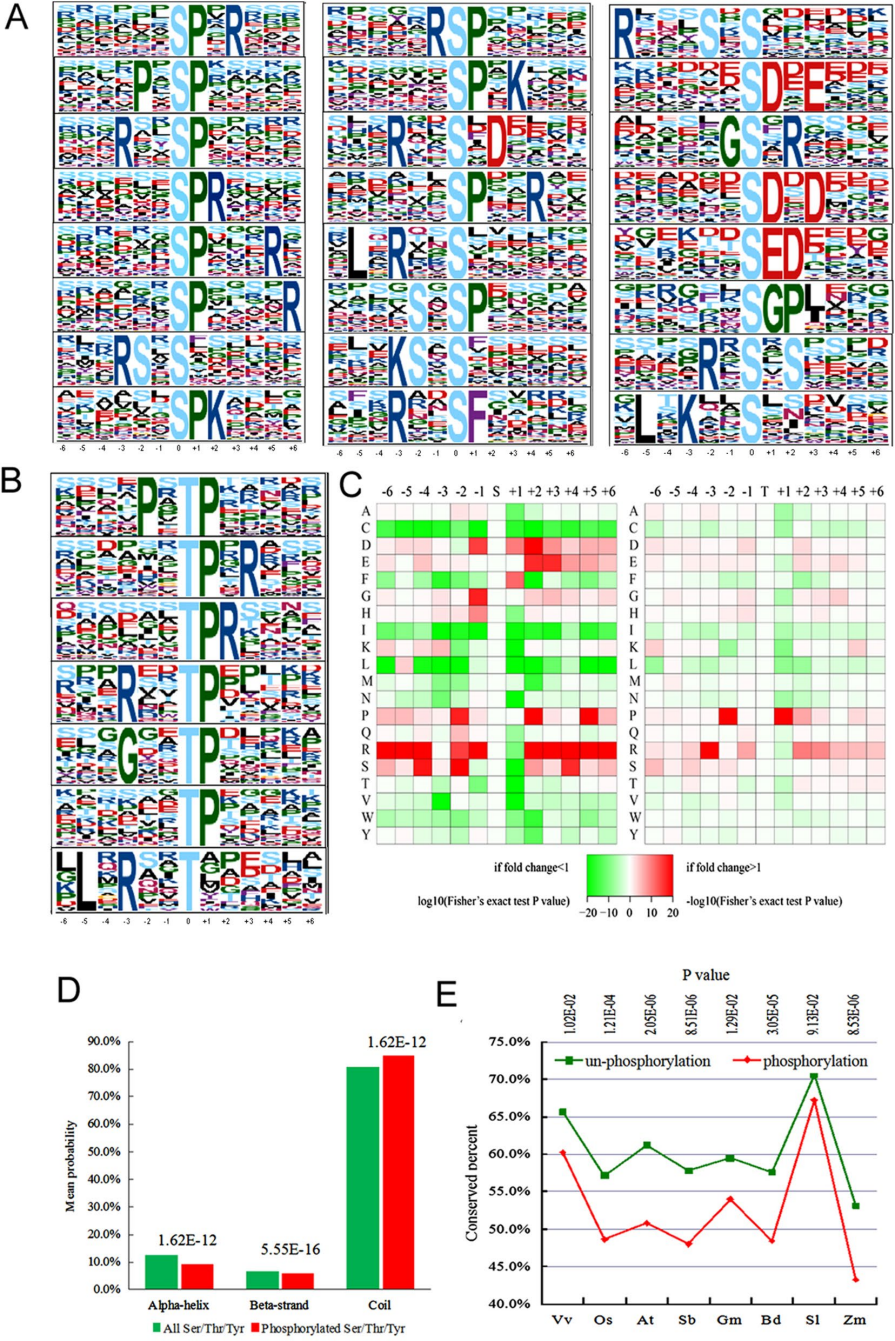
Motif analysis of all the identified phosphosites and sequence properties of the phosphoproteome in petunia. A and B, Phosphorylation motifs and the conservation of phosphorylated sites. The height of each letter corresponds to the frequency of that amino acid residue in that position. Central S (**A**) or T (**B**) refers to phosphorylated Ser/Thr. **C**, Amino acid sequence properties of phosphorylation sites. The heat map shows significant position-specific under- or overrepresentation of amino acids flanking the modification sites. **D**, Predicted protein secondary structures near phosphorylation sites. Probabilities for different secondary structures (coil, α-helix and β-strand) of modified lysines were compared with the secondary structure probabilities of all Ser/thr/Tyr on all proteins identified in this study. **E**, Evolutionary conservation of phosphorylated and nonphosphorylated Ser/thr/Tyr (**F**) on protein orthologues in selected eukaryotic species. Abbreviations: Vv, *Vitis vinifera*; Os, *Oryza sativa* japonica; At, *Arabidopsis thaliana*; Sb, *Sorghum bicolor*; Gm, *Glycine max*; Bd, *Brachypodium distachyon*; Sl, *Solanum lycopersicum*; and Zm, *Zea mays*

To compare the common sequences of phosphopeptides among different plant species, all of the phosphopeptides from this study and from 9 other species in P3DB were used for motif extraction against a background database generated by combining 15,510 random protein sequences derived from the genomes of the ten species. Compared with the phosphorylation motifs detected in the 9 other species, 24 pSer motifs (......SP.R..., ....P.SP....., ...R..SP....., ......SPR...., ......SP...R., ......SP....R, ...RS.S......, ......SPK...., .....RSP....., ......SP.K..., ...R..S.D...., ......SP..R..,. L.R..S......, ....S.SP....., ...KS.S......, ...R..SF....., ...S.S......, ......SD.E..., .....GS.R...., ......SD.D..., ......SED...., ......SGP...., ....R.S.S...., and. L.K..S......) and 7 pThr motifs (....P.TP....., ......TP.R..., ......TPR...., ...R..TP....., ...G..TP....., ......TP.....,. L.R..T......) were identified in our petunia dataset at different abundances (Fig. 5A and B). All identified phosphorylation motifs are listed in Supplementary data Table 7. Among the 31 phosphorylation motifs identified in this study, 2 distinct motifs were found in the PhosphoMotif Finder database:......**S**P...R. and......**S**P....R.

We further analysed the frequencies of neighbouring amino acid residues for phosphorylated serines using iceLogo [31]. In phosphoproteins, hydrophilic residues, such as Glu (E) and Asp, Arg, Ser, and Pro, were significantly preferred in positions adjacent to phosphorylated serines (at positions + 1, + 3, − 1, and − 3) (Fig. 5C). In mammals, neighbouring amino acid residues are often also hydrophilic residues, such as Glu, Arg, Ser, and Pro [32].

The protein secondary structure features were integrated using NetSurfP software [33]. The phosphorylated Ser/Thr/Tyr sites occurred significantly more frequently in unstructured regions of proteins (1.62E-12 for coil) and less often in structured regions (1.62E-12 for α-helix and 5.55E-16 for β-strand) (Fig. 5D).

To study the evolutionary conservation of phosphorylated Ser/Thr/Tyr and nonphosphorylated Ser/Thr/Tyr in plants, petunia proteins with their respective orthologues from 8 other plant species were aligned. Unexpectedly, the results showed that phosphorylated Ser/Thr/Tyr is significantly less conserved than nonphosphorylated Ser/Thr/Tyr, showing that phosphorylated Ser/Thr/Tyr does not maintain stronger selective pressure than nonphosphorylated Ser/Thr/Tyr in plants (Fig. 5E).

### Effect of ethylene treatment on phosphorylation of phosphatases and kinases in petunia corollas

Since ethylene negatively regulates global phosphorylation levels during flower senescence, we further analysed the effect of ethylene treatment on phosphorylation of phosphatases and kinases in petunia corollas. Following the ethylene treatment, 15 phosphosites on 12 phosphatases were downregulated and only 3 phosphosites on 3 phosphatases (Peaxi162Scf00006g03619, Peaxi162Scf00132g00719 and Peaxi162Scf00835g00115) were upregulated (1.685-, 2.407-, and 1.597-fold, respectively); 69 phosphosites on 43 kinases were downregulated and only 10 sites on 9 kinases were upregulated (Supplementary data Table 3).

### Effect of ethylene treatment on the phosphorylation of proteins involved in ethylene biosynthesis and signalling transduction

1-Aminocyclopropane-1-carboxylate (ACC) oxidase (ACO) and ACC synthase (ACS) are two key enzymes that catalyse the last steps of the ethylene biosynthetic pathway. Eleven *ACS* sequences were recovered when the Arabidopsis *AtACS1* sequence was used as a query in BLAST searches of the *Petunia axillaris* draft genome sequence v1.6.2 (https://solgenomics.net/organism/Petunia_axillaris/genome) (Supplementary Fig. S3). In this study, one phosphorylation site of the type 2 ACS protein, PhACS2 (Peaxi162Scf00118g00149, S5), was identified, while no phosphorylation sites of ACO were detected (Supplementary data Table 3).

The phosphosites of 3 ethylene receptors, PhETR2 (Peaxi162Scf00001g34002, S610; Peaxi162Scf00120g00517, T228; Peaxi162Scf00325g00034, S515) (subfamilies 2), were identified and significantly downregulated by more than 2.00-fold by ethylene in vivo. One phosphosite of the raf-like kinase PhCTR1 (Peaxi162Scf00433g00412, S690) was identified. Senen phosphosites in petunia ethylene-insensitive protein 2 (PhEIN2, Peaxi162Scf00123g00089) were identified, and the phosphorylation levels of six sites (S627, S640, S644, S870, S907, and S965) were significantly decreased (2.95-, 8.26-, 3.26-, 1.68-, 3.79-, and 2.40-fold, respectively) after ethylene exposure (Supplementary Fig. S4).

### Effects of ethylene treatment on the phosphorylation of proteins involved in ABA signalling transduction pathways and auxin transport

The ABA signalling component, PP2C, is a major negative regulator of ABA signalling and inhibits SnRK2, a positive regulator of ABA signalling, thus inhibiting activation of the ABA pathway [34]. Here, decreased phosphorylation levels of PP2C (Peaxi162Scf00075g02123, S362, 3.12-fold; Peaxi162Scf00156g00313, S36, 1.50-fold; Peaxi162Scf00529g00005, S71, 1.87-fold; Peaxi162Scf00904g00011, S354, 1.67-fold; Peaxi162Scf00904g00011, S351, 1.80-fold) were identified after ethylene treatment (Supplementary data Table 3).

The phosphorylation levels of both the auxin influx transport protein AUX1 (Peaxi162Scf00141g00048, S34, 2.47-fold; and Peaxi162Scf00014g00614, S14, 1.52-fold) and the auxin efflux transport protein PIN (Peaxi162Scf00159g00068, S215, 2.53-fold; Peaxi162Scf00683g00461, S414, 2.5-fold; and Peaxi162Scf00253g01129, S215, 1.72-fold) decreased after ethylene treatment (Supplementary data Table 3). In addition, the phosphorylation levels of two PhABCBs (Peaxi162Scf00847g00008, S664, 1.6-fold; and Peaxi162Scf00060g00438, S848, 1.67-fold), homologues of the auxin transporter AtABCB1 in Arabidopsis, decreased following ethylene treatment. These results showed that ethylene played an important role in auxin transport, including in both influx and efflux.

### Effects of ethylene treatment on the proteins involved in remobilization of essential nutrients

During flower senescence, the programmed degradation of macromolecules and remobilization of essential nutrients have been observed [1]. The phosphorylation levels of sugar transporter ERD6 (Peaxi162Scf00095g00120, S30, 3.65-fold), potassium transporter 7 (Peaxi162Scf00001g00018, S80, 1.86-fold; Peaxi162Scf00003g03544, S167, 2.305-fold; and Peaxi162Scf00806g00011, S194, 2.35-fold; S195, 1.79-fold), transmembrane amino acid transporter (Peaxi162Scf00040g00315, S22, 3.88-fold; and Peaxi162Scf00076g00615, S54, 2.68-fold), sugar transporter ERD6 (Peaxi162Scf00095g00120, S30, 3.65-fold), and ammonium transporter 1 (Peaxi162Scf00125g00122, S463, 1.82-fold) increased during ethylene-mediated senescence in petunia corollas (Supplementary data Table 3). These results suggested that a different nutrient remobilization programme operates during ethylene-mediated senescence.

### Ethylene treatment decreases the phosphorylation levels of proteins involved in RNA metabolism

To our knowledge, the effect of ethylene treatment on alternative splicing (AS) has not been reported in plants. SR and hnRNP proteins are alternative splicing regulators. Phosphorylation regulation of SR proteins plays an important role in spliceosome assembly and in regulating SR protein recycling in the cell [35]. In this study, numerous phosphoproteins involved in RNA metabolism, including components of RNA transport proteins, mRNA surveillance, and the spliceosome, were identified (Supplementary Fig. S5). A total of 47 phosphosites in 18 proteins involved in the spliceosome were downregulated by ethylene, including 15 sites in 3 serine/arginine-rich splicing factors (SRs), including SFRS2 (Peaxi162Scf00041g01416 and Peaxi162Scf00152g01021) and RSZ21 (Peaxi162Scf00213g00074) (Supplementary Fig. S5A). Nine SR proteins were detected and extensively phosphorylated at serine residues in their serine/arginine-rich domains (Supplementary data Table 4). Eight phosphosites in three ATP-dependent RNA helicases (Peaxi162Scf00287g01631, S31, 1.99-fold; Peaxi162Scf00177g00220, S42, 2.38-fold) were downregulated by ethylene. The fold changes of these identified phosphosites ranged between 5.00 and 1.58. These results suggested that phosphorylation of the homologues might also affect splicing in petunia.

In addition to the arginine/serine-rich protein mentioned above, another eight phosphoproteins involved in RNA transport were identified (Supplementary Fig. S5C), including 6 translation initiation factors (Peaxi162Scf00587g00036, Peaxi162Scf00774g00012, Peaxi162Scf00249g00716, Peaxi162Scf00793g00038, Peaxi162Scf00401g00035, and Peaxi162Scf00104g00187) and one protein arginine N-methyltransferase (Peaxi162Scf00045g01452).

### Ethylene treatment increases the number of AS precursor RNAs in petunia corollas

**Fig. 6:**
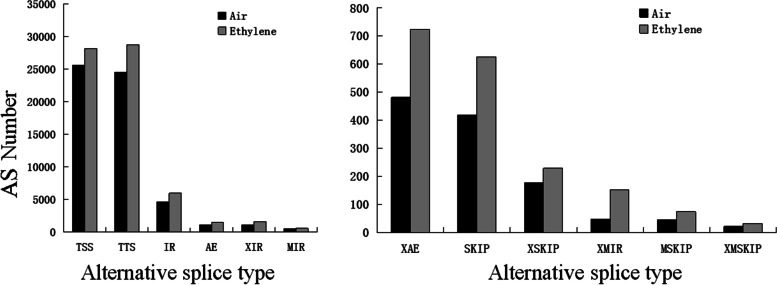
Effect of ethylene treatment on alternative splicing. Abbreviations: AE, Alternative exon ends (5′, 3′, or both); IR, Intron retention (IR_ON, IR_OFF pair); MIR, Multi-IR (MIR_ON, MIR_OFF pair); MSKIP, Multi-exon SKIP (MSKIP_ON, MSKIP_OFF pair); SKIP, Skipped exon (SKIP_ON, SKIP_OFF pair); TSS: Alternative 5′ first exon (transcription start site); TTS: Alternative 3′ last exon (transcription terminal site); XAE, Approximate AE; XIR, Approximate IR (XIR_ON, XIR_OFF pair); XMIR, Approximate MIR (XMIR_ON, XMIR_OFF pair); XMSKIP, Approximate MSKIP (XMSKIP_ON, XMSKIP_OFF pair); XSKIP, Approximate SKIP (XSKIP_ON, XSKIP_OFF pair)

Since ethylene treatment decreases the phosphorylation levels of proteins involved in the spliceosome, we further analysed the effect of ethylene treatment on the AS of mRNAs. We previously obtained transcriptome data of petunia corollas treated with 16 h air and 2 μl l^− 1^ ethylene. We analysed 12 types of AS, including approximate AE (XAE), alternative exon ends (5′, 3′, or both, AE), approximate MIR (XMIR_ON, XMIR_OFF pair, XMIR), multi-IR (MIR_ON, MIR_OFF pair, MIR), approximate IR (XIR_ON,XIR_OFF pair, XIR), intron retention (IR_ON, IR_OFF pair, IR), approximate MSKIP (XMSKIP_ON,XMSKIP_OFF pair, XMSKIP), multi-exon SKIP (MSKIP_ON,MSKIP_OFF pair, MSKIP), approximate SKIP (XSKIP_ON, XSKIP_OFF pair, XSKIP), skipped exon (SKIP_ON, SKIP_OFF pair, SKIP), alternative 3′ last exon (transcription terminal site, TTS) and alternative 5′ first exon (transcription start site, TSS). The results showed that the ethylene treatment increased the total number of all types of alternative splicing of mRNA (Fig. 6; Supplementary data Tables 8 and 9, air and treatment, respectively), which represents the first report of this effect. In the proteome of corollas treated with 16 h air and 2 μl l^− 1^ ethylene, differential proteins were not enriched in the spliceosome pathway while the proteins with downregulated phosphorylation levels were enriched in the spliceosome pathway, which showed the important role of phosphorylation on the AS of mRNAs.

### Ethylene treatment increases the number of AS precursor RNAs of genes involved in ethylene biosynthesis and signalling transduction in petunia corollas

Next we analysed the effect of ethylene treatment on the number of AS precursor RNAs of genes involved in ethylene biosynthesis and signalling transduction. The results showed that there were AS events in 4 *PhACSs*, 5 *PhACOs*, 7 *ethylene receptors*, 2 *PhCTRs*, 1 *PhEIN2* and 2 *PhEILs* in air- and ethylene-treated petunia corollas. Ethylene treatment increased the number of AS events of precursor RNAs of most genes involved in ethylene biosynthesis and signalling transduction (Table 1 and Supplementary data Table 10). There were 2, 2, 2 and 8 AS events from *PhACS8*, *PhACO3*, *PhETR2E* and *PhCTR1* in ethylene-treated corollas, respectively, while there was no AS event in these genes in air-treated corollas.

**Table 1:** Effect of ethylene treatment on the AS number of the genes involved in ethylene biosynthesis and signalling transduction

Gene	Gene ID	AS number	
		Air treatment	Ethylene treatment
***PhACS5***	Peaxi162Scf00822g00212	1	1
***PhACS6***	Peaxi162Scf00192g00920	2	5
***PhACS7***	Peaxi162Scf00102g01634	1	2
***PhACS8***	Peaxi162Scf00074g01725	0	2
***PhACO1***	Peaxi162Scf01333g10016	2	4
***PhACO2***	Peaxi162Scf00521g00613	1	3
***PhACO3***	Peaxi162Scf00141g00330	0	2
***PhACO4***	Peaxi162Scf01333g10015	2	5
***PhACO5***	Peaxi162Scf00047g01927	1	4
***PhETR1A***	Peaxi162Scf00111g00820	5	4
***PhETR1B***	Peaxi162Scf00000g00584	3	4
***PhETR2A***	Peaxi162Scf00325g00034	4	5
***PhETR2B***	Peaxi162Scf00001g34002	6	3
***PhETR2C***	Peaxi162Scf00120g00517	2	2
***PhETR2D***	Peaxi162Scf00519g00115	2	2
***PhETR2E***	Peaxi162Scf00024g00157	0	2
***PhCTR1***	Peaxi162Scf00433g00412	0	8
***PhCTR2***	Peaxi162Scf00110g02024	15	17
***PhEIN2***	Peaxi162Scf00123g00089	4	11
***PhEIL1***	Peaxi162Scf00559g00420	2	3
***PhEIL4***	Peaxi162Scf00593g00325	1	0

We further examined the effect of ethylene treatment on the AS events in *PhACS8*, *PhACO3*, *PhCTR1* and *PhEIN2* by PCR assay. The results showed that there were more bands using the cDNA from ethylene-treated corollas as template than that from air-treated corollas as template in the selected AS events in *PhACS8*, *PhACO3*, *PhCTR1* and *PhEIN2* (Supplementary Fig. S6), which showed ethylene treatment increased the number of AS precursor RNAs of these genes.

### Correlation between the global phosphoproteome and ubiquitylome

Ubiquitination plays an important role in protein degradation, and phosphorylation controls the biological activity of proteins and plays a pivotal role in many biochemical processes [36]. In this study, and our previous work [22], 1184 phosphorylated proteins and 1515 ubiquitylated proteins were identified in petunia corollas following ethylene or air treatment, respectively. A total of 291 (12.1% of phosphorylation and ubiquitylation proteins) proteins were both phosphorylated and ubiquitylated (Supplementary Fig. S7, Supplementary data Table 11). In addition, global ubiquitination levels increase [22] while global phosphorylation levels decrease during senescence, which could indicate the involvement of protein phosphorylation and ubiquitination in senescence in a concerted manner.

## Discussion

In this study, quantitative investigation of the petunia phosphoproteome following ethylene or air treatment showed that ethylene negatively regulates global phosphorylation levels and that phosphorylation of many proteins may not be necessary during flower senescence. We attempted to analyse the cause of the decrease in phosphorylation levels following ethylene treatment and discussed the effect of ethylene treatment on the phosphorylation of proteins related to senescence.

### Effect of ethylene treatment on the abundance of proteins and phosphorylation of phosphatases and kinases in petunia corollas

Protein phosphorylation regulates many biological processes, such as cell division, metabolism, and intracellular signal transduction, and plays a key role in plant growth and development [37]. The expression of many protein kinases and phosphatases is changed during ageing of Arabidopsis, and several kinases and phosphatases have shown a key role in regulating leaf senescence [17, 21, 22]. Generally, kinases may be more required than phosphatases [16]. In this study, however, during ethylene-mediated flower senescence, the global phosphorylation level significantly decreased, which shows that the high phosphorylation levels of most proteins are no longer required, dephosphorylation occurs for most expressed proteins in corollas and the activities of the proteins are decreased during senescence because maintaining high phosphorylation levels of proteins is energetically costly. Our results showed that phosphatases may be required in these processes.

The phosphorylation analysis in this study is performed parallel to the proteome and ubiquitylome analysis of our previous research [28]. In the proteome, 5 kinases were enriched in downregulated proteins. Only one phosphatase (Peaxi162Scf00845g00034) was enriched in upregulated proteins [28], Supplementary data Table 1). In the phosphoproteome, 15 and 3 phosphosites on 12 and 3 phosphatases were downregulated and upregulated, respectively; however, 69 phosphosites on 43 kinases were downregulated and only 10 sites on 9 kinases were upregulated. Based on these data, the decreased level of ethylene-mediated phosphorylation could be attributed to the upregulated phosphatase at the protein level, the downregulated phosphosites in kinases, which resulted in decreased kinase activity, or the upregulation of 3 phosphosites on 3 phosphatases, which increased phosphatase activity. In addition, previous studies showed that Arabidopsis protein phosphatases SAG113 and MKP2 positively regulated leaf senescence [24–26].

### Ethylene regulates ethylene biosynthesis and signalling transduction pathways via phosphorylation

In this study, ethylene appeared to affect ethylene signal transduction pathways. The phosphorylation levels of some components of ethylene signal transduction were changed by ethylene treatment (Supplementary data Table 3).

ACO and ACS have been extensively studied in petunia flowers [38–41]. In apple fruits, the abundance of two ACOs was significantly increased [42]. In the *Medicago truncatula skl* mutant, an ethylene-insensitive legume mutant, the ACO protein was significantly upregulated compared with the wild-type control [43]. ACS is a very important enzyme for petunia petal senescence [44–50]. PhFBH4 silencing or overexpression reduced or increased the mRNA levels of PhACS1 and PhACO1, respectively, and influenced ethylene production [44]. Based on distinct consensus sequences present in the C-termini, the ACS isoforms can be grouped into three types (types 1–3) [51]. In tomato, the type 1 and 2 ACS proteins SlACS2 and SlACS3, respectively, have sites for phosphorylation while the type 3 ACS protein lacks sites for phosphorylation [52]. In petunia, three *ACSs* were cloned [44]. Here, only one phosphorylation site of PhACS2 was identified. In Arabidopsis, the type 1 ACS proteins ACS2 and ACS6 have been shown to be phosphorylated by the mitogen-activating protein kinase (MAPK) MPK6 [53] or by calcium-dependent protein kinases (CDPKs) [54], and phosphorylation of ACS6 enhances the stability of ACS proteins [55]. The abundance of both proteins and phosphonates of ACS family proteins were not significantly changed (Supplementary data Table 3) [28]. The phosphorylation of PhACS2 plays an important role during corolla senescence. Whether the phosphorylation of PhACS2 enhances the stability of ACS proteins needs further study.

The ethylene receptor gene family can be divided into subfamilies 1 and 2. In Arabidopsis, all five ethylene receptor proteins autophosphorylate in vitro [56]. However, in tomato plants, treatment of preclimacteric fruits with ethylene resulted in accumulation of LeETR4 and Never-ripe fruits with reduced phosphorylation [57]. In this study, the phosphosites of 3 PhETR2s were significantly downregulated after ethylene exposure. Autokinase activity of the purified receptor is also controlled by ethylene [58]. These results suggested that the effect of ethylene treatment on the phosphorylation of ethylene receptors depends on the species or tissue. Alterations in the phosphorylation states of receptors are likely to be an initial response upon ethylene binding [57].

Phosphoproteins were also identified for CTR1, a negative regulator of ethylene signalling in Arabidopsis [59]. In this study, one phosphosite of PhCTR1 was identified. Further investigation is required to understand the function of the phosphorylation of PhCTR1.

EIN2 acts downstream of ethylene receptors and upstream of EIN3/EIL and plays an important role in regulating flower senescence [60]. CTR1 interacts with and directly phosphorylates the cytosolic C-terminal domain of EIN2. Mutations that block EIN2 phosphosites result in nuclear localization of the EIN2 C-terminus [61]. Mass spectrometry analysis of the kinase reaction identified six phosphosites in Arabidopsis EIN2. Of these, CTR1 phosphorylates EIN2 in vivo on at least four sites [61, 62]. In the current work, the phosphorylation levels of six sites on PhEIN2 were significantly decreased after ethylene exposure. In the absence of ethylene, phosphorylation of Arabidopsis EIN2 by CTR1 has been shown to prevent the ethylene signal from reaching downstream transcription factors [61], and our results support this opinion.

The results mentioned above showed that the phosphorylation levels in the ethylene signalling pathway can be regulated by ethylene. These findings significantly promote our understanding of ethylene signalling transduction mechanisms (Supplementary Fig. S4).

### Ethylene regulates auxin transport via phosphorylation level

Phosphorylation activates many components of the auxin efflux (but not influx) system [63, 64]. In this study, the phosphorylation levels of both AUX1 and PIN decreased after ethylene treatment. Thus, it appears that decreases in phosphorylation of both efflux and influx carriers weakened auxin transport in petunias. In addition, the phosphorylation levels of two PhABCBs decreased following ethylene treatment. These results showed that ethylene played an important role in auxin transport, including in both influx and efflux.

### Regulation of ethylene on the number of AS precursor RNAs of genes involved in ethylene biosynthesis and signalling transduction in petunia corollas

Previous studies suggested that ethylene treatment promote ethylene autocatalysis and accelerated flower senescence in petunia and carnation [65]. In addition, ethylene treatment changed the expression of genes inveolved in ethylene biosynthesis and signalling transduction [28, 66]. In this study, the results of both transcriptome and PCR assay showed that ethylene treatment increased the number of AS precursor RNAs of most genes involved in ethylene biosynthesis and signalling transduction in petunia corollas, which could provide a new perspective of regulation of ethylene on flower senescence in petunia.

## Conclusion

In this study, a global analysis of phosphoproteome regulation by ethylene and further insights into the dynamics of individual phosphosites were provided. Our results revealed the negative regulation of global phosphorylation by ethylene and showed that protein dephosphorylation may play an important role in ethylene-induced corolla senescence in petunia. Our results revealed the important role of phosphorylation in hormone signal transduction and transport, ion transmembrane transport and remobilization of essential nutrients during ethylene-induced senescence. For the first time, our results show that the spliceosome pathway was enriched in proteins with downregulated sites, indicating the involvement of phosphorylation in RNA metabolism during ethylene-induced senescence. The provided data set may serve as an important resource for the functional analysis of phosphorylation of serine, threonine and tyrosine and facilitate a better understanding of the senescence process in this model petunia.

## Methods

### Plant material

The seeds of petunia ‘Mitchell’ were acquired from the lab of Manzhu Bao (Huazhong Agricultural University). Petunia ‘Mitchell’ plants were grown under greenhouse conditions (22–25 °C) [4]. To prevent self-pollination, petunia flowers were emasculated one day before they were fully open. Eight to 10 flowers were harvested at the anthesis stage (corollas 90° reflexed) and then put in tap water immediately. All tissues were frozen in liquid nitrogen and stored at − 80 °C until use for RNA extraction. Three biological replicates were performed. All methods were carried out in accordance with relevant guidelines and regulations.

### Ethylene treatment

Petunia flowers were treated with ethylene according to our previously defined protocols [67]. Flowers were harvested at anthesis, put in flasks with distilled water and subsequently treated with 2 μl l^− 1^ ethylene (treatment group) and air (control group) for 16 h. Then, corollas from 8 to 10 flowers were collected, frozen in liquid nitrogen, and stored at − 80 °C for the following experiments. Three biological replicates were performed.

### Protein extraction

Protein extraction was performed according to the method of Guo et al. [28]. The corolla of *Petunia hybrida* was ground in liquid nitrogen, and then the cell powder was transferred to a centrifuge tube and sonicated three times on ice in lysis buffer with a high-intensity ultrasonic processor (Scientz). Petunia corollas were ground in liquid nitrogen, and then the cell powder was transferred to a centrifuge tube and sonicated three times on ice in lysis buffer (0.1% Protease Inhibitor Cocktail, 1% Triton-100, 65 mM DTT and 8 M urea) with a high-intensity ultrasonic processor (Scientz). The protein was precipitated with 15% TCA at − 20 °C for 2 h. The supernatant was discarded after centrifugation for 10 min at 4 °C. The remaining precipitate was washed with cold acetone 3 times. The protein was redissolved in buffer (100 mM TEAB, 8 M urea, pH 8.0). Three biological replicates were performed.

### Trypsin digestion

Trypsin digestion was performed according to the method of Guo et al. [28]. The protein sample was diluted by adding 100 mM TEAB to urea concentrations less than 2 mol l^− 1^. During the first digestion overnight, trypsin was added at a mass ratio of 1:50 trypsin to protein, and in the second 4-h digestion, the mass ratio of trypsin to protein was 1:100. Approximately 100 μg protein was digested with trypsin for each sample.

### TMT labelling and HPLC fractionation

TMT labelling and HPLC fractionation were performed according to the method of Guo et al. [28].

### Affinity enrichment

To enrich phosphopeptides, the peptide mixtures were incubated with a vibrating suspension of IMAC microspheres. The IMAC microspheres with enriched phosphopeptides were collected, and the supernatant was removed. The IMAC microspheres were washed with 50% ACN/6% TFA and 30% ACN/0.1% TFA sequentially to remove nonspecifically adsorbed peptides. Elution buffer was added, and the enriched phosphopeptides were eluted with vibration to elute the enriched phosphopeptides from the IMAC microspheres. The supernatant containing phosphopeptides was collected and lyophilized for LC-MS/MS analysis.

### LC-MS/MS analysis

LC-MS/MS analysis was performed according to the method of Guo et al. [28]. Three biological replicates were performed.

### Database search

The resulting MS/MS data were processed using the MaxQuant search engine (v.1.5.2.8) [68]. Tandem mass spectra were searched against the petunia genome (https://solgenomics.net/organism/Petunia_axillaris/genome). Trypsin/P was specified as a cleavage enzyme, allowing up to 2 missed cleavages, 4 modifications per peptide and 5 charges. Phosphorylation on serine, threonine and tyrosine was specified as a fixed modification.

### Bioinformatic analysis

Bioinformatic analysis was performed according to previously described protocols [28, 69]. The two-tailed t-test was applied to calculate the false discovery rate (FDR) for significant differences. Phosphosites of Eth16/Air16 with ratios > 1.5 and FDR < 0.05 were regarded as upregulated, while phosphosites with ratios < 1/1.5 and FDR < 0.05 were regarded as downregulated.

### Enrichment of gene ontology and pathway analysis

Proteins were classified by gene ontology (GO) annotation into three categories: biological process, cellular compartment and molecular function. For each category, a two-tailed Fisher’s exact test was employed to test the enrichment of the differentially modified protein against all identified proteins. The GO with a corrected *p* value < 0.05 was considered significant.

The KEGG database (v.2.5, http://www.kegg.jp/kegg/mapper.html) was used to identify enriched pathways by a two-tailed Fisher’s exact test to test the enrichment of the differentially modified protein against all identified proteins (81). The pathway with a corrected *p* value < 0.05 was considered significant. These pathways were classified into hierarchical categories according to the KEGG website.

### Phosphorylated protein secondary structure analysis

The secondary structure of phosphorylated proteins was determined according to the method of Guo et al. [28]. We showed the distribution of phosphorylated and nonphosphorylated amino acids in protein secondary structures in this study. Probabilities for three secondary structures, alpha-helix, beta-strand and coil, of phosphorylated Ser/Thr/Tyr were compared with those of all Ser/Thr/Tyr in all proteins identified. The local secondary structures of proteins were further investigated using NetSurfP.

### Conservation analysis of phosphorylated proteins

A conservation analysis of the phosphorylated proteins was performed according to the method of Guo et al. [28]. First, by blasting against eight species in the UniProtKB database, namely, *Vitis vinifera, Solanum lycopersicum*, *Glycine max*, *Arabidopsis thaliana*, *Zea mays, Sorghum bicolor*, *Brachypodium distachyon*, and *Oryza sativa* ‘japonica’, proteins homologous to the phosphorylated protein identified in this study were obtained. The homologous proteins of the phosphorylated proteins in this study were obtained using the sequence alignment software BLASTp. The multiple sequence alignment software muscle was used to align homologues to find conserved sites.

### Prediction of alternative splicing (AS) of precursor mRNA

An RNA-seq analysis was conducted using StringTie v.1.3.3 [70]. The alternative splicing events in our previous sample [4] were classified and counted using Asprofile (v1.0.4) software [71]. The Venny online tool (http://bioinfogp.cnb.csic.es/tools/venny/index.html) was used for the statistics of the number of genes with alternative splicing.

### PCR assay of AS events of genes involved in ethylene biosynthesis and signalling transduction

Specific primers (Supplemental Table S1) were designed at about 60 bp away from splice site sequences of the AS events of genes involved in ethylene biosynthesis and signalling transduction. PCR was performed using the cDNA of air- and ethylene-treated corollas. Three biological repeats, each including three technical repeats, were performed for each experiment.

## Supplementary Information

**Additional file 1.**

**Additional file 2.**

**Additional file 3.**

**Additional file 4.**

**Additional file 5.**

**Additional file 6.**

**Additional file 7.**

**Additional file 8.**

**Additional file 9.**

**Additional file 10.**

**Additional file 11.**

**Additional file 12.**

## Data Availability

Sequence data from this article can be found in the GenBank/EMBL data libraries under accession number FN014209 (petunia ACTIN). Sequence data from this manuscript can be found in GenBank (The following secure token has been created to allow review of record GSE136921 while it remains in private status: yxifsqkyxxstbmp). The mass spectrometry proteomics data have been deposited to the ProteomeXchange Consortium via the PRIDE (Perez-Riverol et al., 2019) partner repository with the dataset identifier PXD005599 (Reviewer account details: Username: reviewer69405@ebi.ac.uk, Password: 6POtxHak).

## Abbreviations

ABA: Abscisic acid
ABCB: ATP binding cassette subfamily B
ACN: Acetonitrile
ACO: 1-Aminocyclopropane-1-carboxylate oxidase
ACS: 1-Aminocyclopropane-1-carboxylate synthase
AS: Alternative splicing
AUX: Auxin
CTR: Constitutive triple response
eIF: Eukaryotic initiation factor
EIL: EIN3-like
EIN2: Ethylene insensitive 2
EIN3: Ethylene insensitive3
ERD: Early-responsive to dehydration
ETR: ETHYLENE receptor
FDR: False discovery rate
GO: Gene ontology
hnRNP: Heterogeneous nuclear ribonucleoprotein
HPLC: High performance liquid chromatography
IMAC: Immobilized metal affinity chromatography
iTRAQ: Isobaric tags for relative and absolute quantitation
KEGG: Kyoto encyclopaedia of genes and genomes
LC-MS/MS: Liquid chromatograph-mass spectrometer
MKK: Mitogen-activated protein kinase kinase
MPK: Mitogen-activated protein kinase
PP2C: Protein phosphatase type 2C
PRIDE: Proteomics identifications
PT: a Putative phosphate transporter
PTM: Post-translational modifications
RSZ: Arginine/serine(RS)-rich domain harbour a Zn knuckle motif
SFRS: Serine/arginine-rich (SR) family of splicing factor
SLU: Synergistic lethal with U5 snRNA
SR: Splicing regulator
TFA: Trifluoroacetic acid
TMT: Tandem mass tag
