# Supplementary material for: Phosphoproteome analysis reveals the involvement of protein dephosphorylation in ethylene-induced corolla senescence in petunia
Source: BMC Plant Biol. 2021 Nov 3;21:512. doi: 10.1186/s12870-021-03286-x (PMC8565076; doi:10.1186/s12870-021-03286-x)
Supplement: Supplementary file 12 — Additional file 12. [file 12870_2021_3286_MOESM12_ESM.doc]

Supplemental Table S1 Primer sequences of genes used in verification of alternative splicing in single events.

| Gene | Event type | Event id | Forward primer (5′→3′) | Reverse primer (5′→3′) |
| --- | --- | --- | --- | --- |
| *PhACS8* | TTS | 1010103 | GGAGGAGCAACTGGAGCTCATGA | AATAGCTTTGCTAATTCCCTTTGT |
| *PhACO3* | TTS | 1015601 | AAGCGCATTCTTTGTTTGGCA | GAGGGCATTGAGGATACATTG |
| *PhCTR1* | SKIP_ON | 1030027 | AGAGCTGATGCTTCCTCATGTC | TCTCTCCATGTATCTATCAGC |
| *PhCTR1* | TTS | 1027689 | GGATTGTATAGGATCGATCGTG | GAGATAGAAGATGGGCCATTG |
| *PhEIN2* | AE | 1013885 | TCAGTGGAGAGAGTGCATTTG | CAACTGCTTGTCTACCAAGAT |

|  | **Pearson correlation coefficient** | | | | | |
| --- | --- | --- | --- | --- | --- | --- |
|  | Air16-1 | Air16-2 | Air16-3 | Eth16-1 | Eth16-2 | Eth16-3 |
| Air16-1 | 1 | 0.877699429 | 0.674341297 | -0.763868923 | -0.765578945 | -0.805399385 |
| Air16-2 | 0.877699429 | 1 | 0.817782249 | -0.809599574 | -0.826375664 | -0.818466162 |
| Air16-3 | 0.674341297 | 0.817782249 | 1 | -0.679493725 | -0.744941671 | -0.656797811 |
| Eth16-1 | -0.763868923 | -0.809599574 | -0.679493725 | 1 | 0.94074636 | 0.92002928 |
| Eth16-2 | -0.765578945 | -0.826375664 | -0.744941671 | 0.94074636 | 1 | 0.926103268 |
| Eth16-3 | -0.805399385 | -0.818466162 | -0.656797811 | 0.92002928 | 0.926103268 | 1 |

**Supplementary Figure S1** Repeatability test between samples in the phosphoproteome of petunia corollas treated with air or 2 µl l^-1^ ethylene for 16 h.

A

B


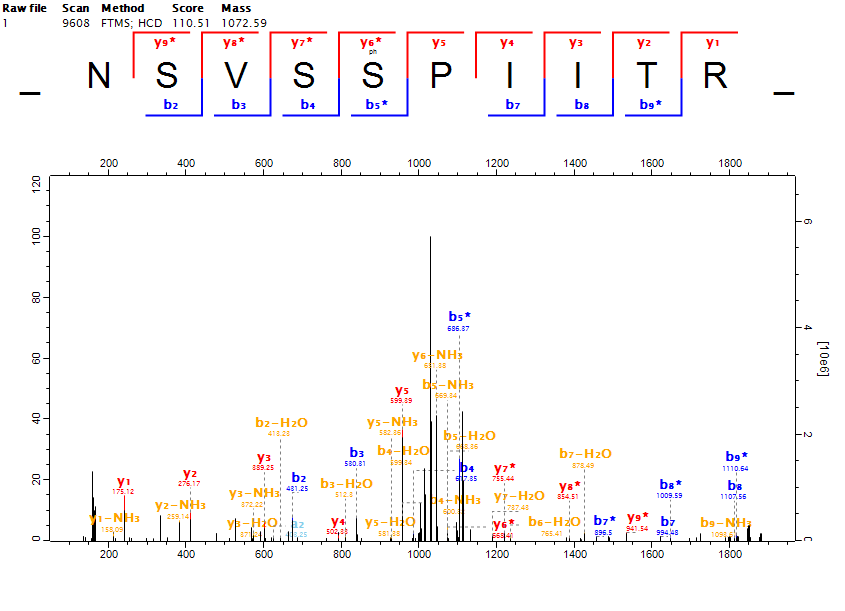


ph

C

D


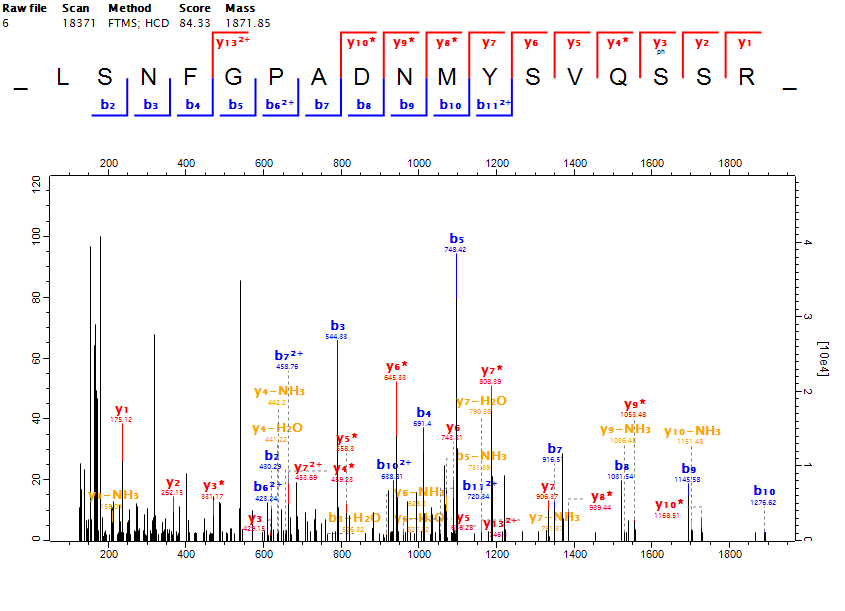

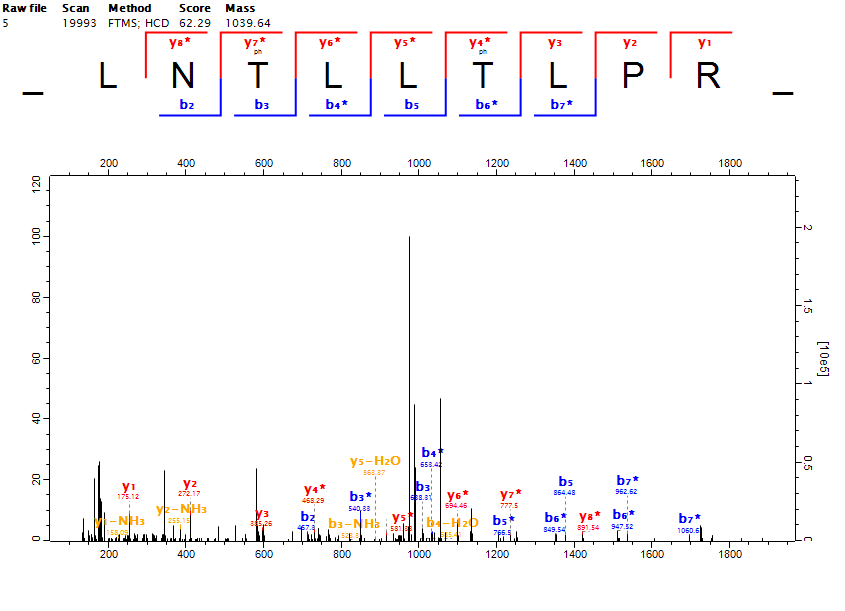


ph

ph

**Supplementary Figure S2** MS/MS spectra for the phosphorylation of several proteins. A, Ethylene receptor PhETR2 (Peaxi162Scf00120g00517.1, T228); B, ABC transporter B (Peaxi162Scf00847g00008.1, S664) phosphorylation; C, Protein phosphatase 2C (Peaxi162Scf00529g00005.1, T503); and D, Auxin efflux facilitator 4 (Peaxi162Scf00159g00068.1, S276).

Type 3

Type 2

Type 1

P

**Supplementary Figure S3** Phylogenetic tree of ACSs. Four petunia PhACSs (PhACS1, Peaxi162Scf00020g00149; PhACS2, Peaxi162Scf00118g00149; PhACS3, Peaxi162Scf00096g01846; PhACS4, Peaxi162Scf00381g00219 [Sol Genomics Network]) were aligned with the Arabidopsis ERF family (AtACS1, AT3G61510; AtACS2, AT1G01480; AtACS4, AT2G22810; AtACS5, AT5G65800; AtACS6, AT4G11280; AtACS7, AT4G26200; AtACS8, AT4G37770; AtACS9, AT3G49700; AtACS11, AT4G08040 [GenBank]) and five *Solanum lycopersicum* SlACSs (SlACS1, Solyc08g081535; SlACS2, Solyc01g095080; SlACS3, Solyc02g091990; SlACS4, SlACS5, Solyc04g077410 [Sol Genomics Network]).

**Supplementary Figure S4** Ethylene signalling transduction remodelling in the presence of ethylene. Differentially expressed proteins are framed in oval boxes, and differentially phosphorylated proteins have round boxes. The green box indicates upregulation, and the blue box indicates no significant changes upon ethylene treatment. Abbreviations: P, phosphorylation;. Cb5, cytochrome b5-like; CTR, constitutive triple response; EIN, ethylene insensitive; EIN2-C, EIN2 C end; ERS, ethylene response sensor; ETR, ethylene resistant; RTE1, REVERSION-TO-ETHYLENE SENSITIVITY1.


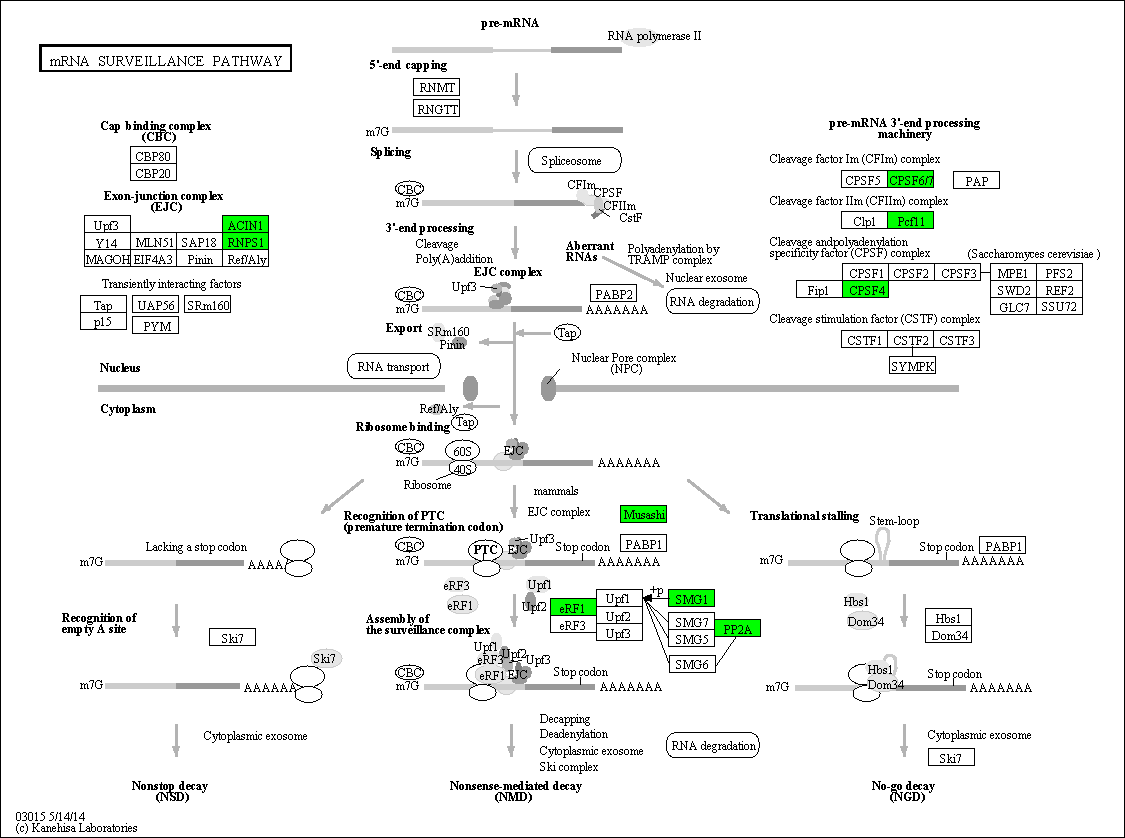

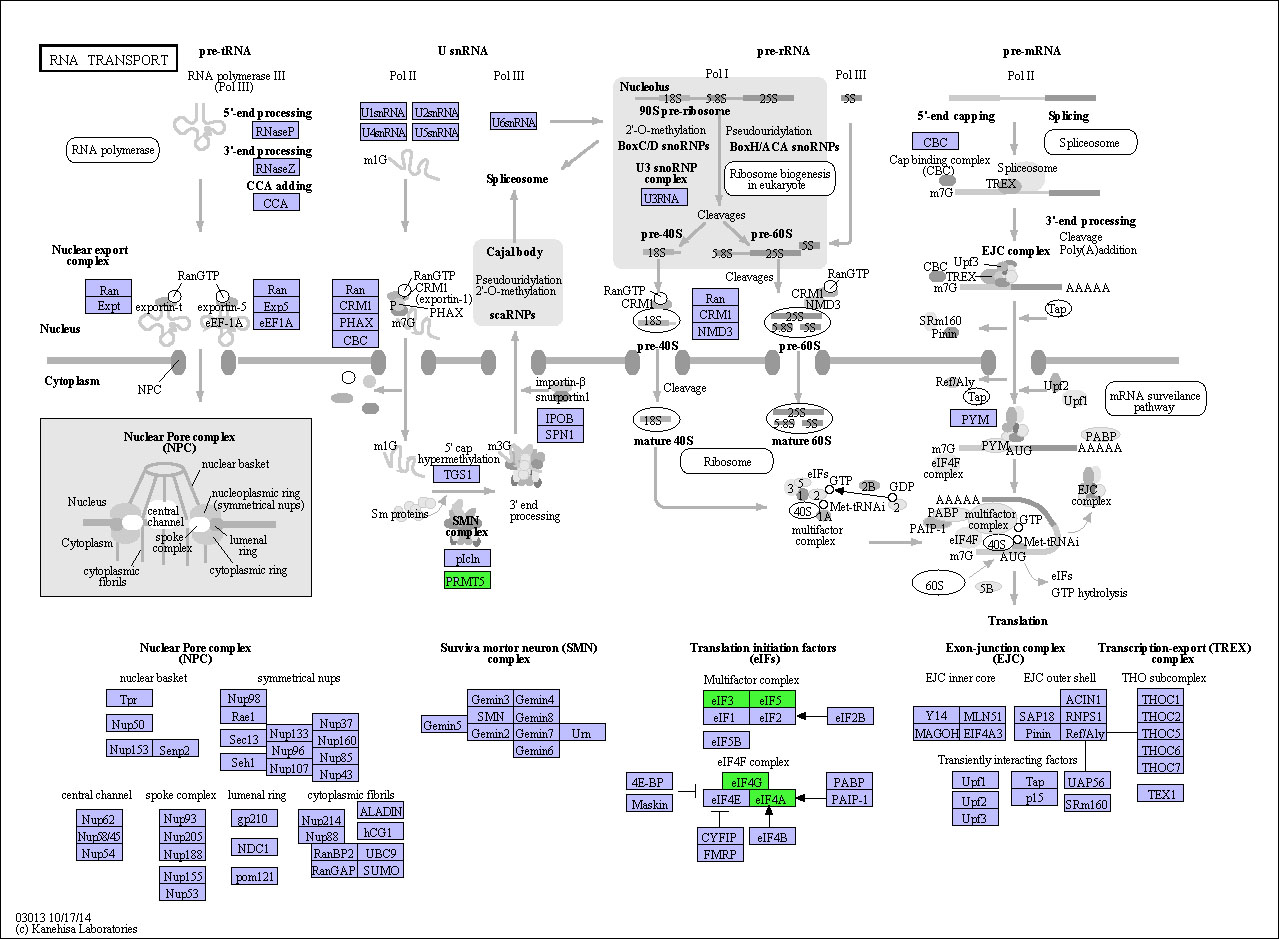


B

A

C


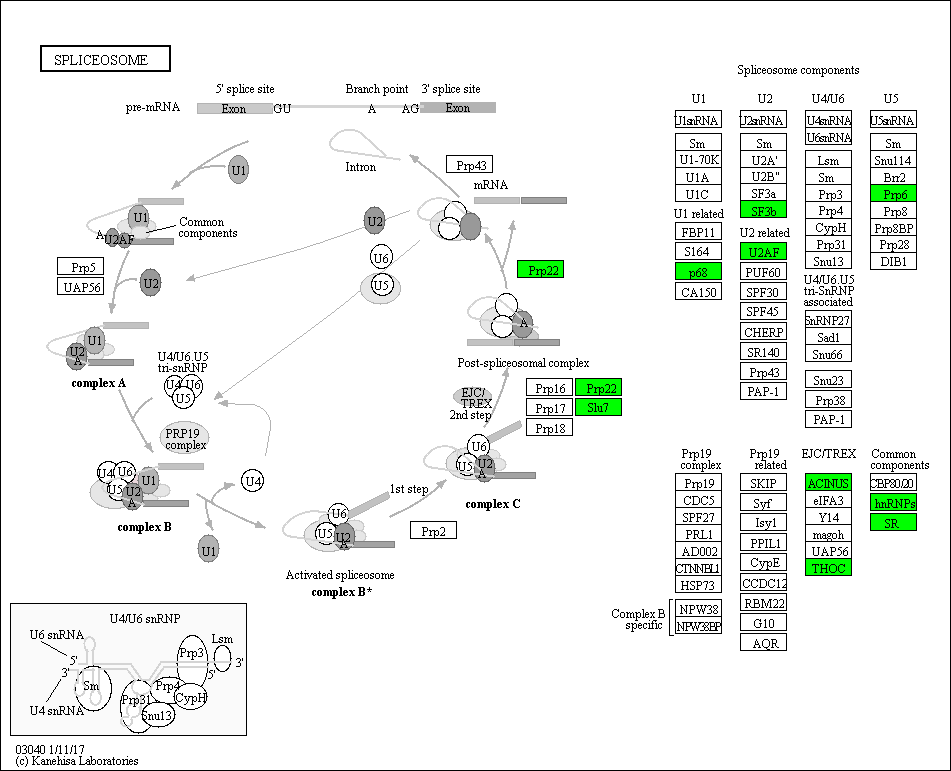


**Supplementary Figure S5** Effects of ethylene on RNA metabolism in petunia. A, Effects of ethylene on the spliceosome (KEGG Pathway (www.kegg.jp/kegg/kegg1.html): ko03040); B, Effects of ethylene on mRNA surveillance (KEGG Pathway: ko03015); C, Effects of ethylene on RNA transport (KEGG Pathway: ko03013). The green box indicates downregulation in phosphorylation level upon ethylene treatment.


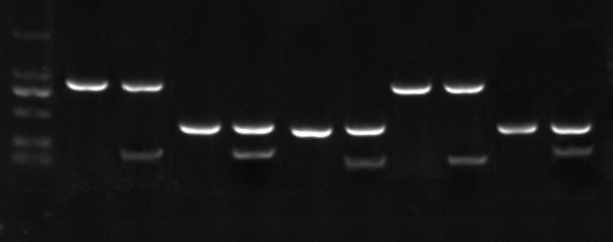


M 1 2 3 4 5 6 7 8 9 10

Air Eth Air Eth Air Eth Air Eth Air Eth

**Supplementary Figure S6** Effect of ethylene treatment on AS events in the genes involved in ethylene biosynthesis and signaling in petunia. Lane M, Maker; Lane 1 and 2, *PhACS8* (Event id 1010103 of event type TSS); Lane 3 and 4, *PhACO3* (Event id 1015601 of event type TSS); Lane 5 and 6, *PhCTR1* (Event id 1030027 of event type SKIP_ON); Lane 7 and 8, *PhCTR1* (Event id 1027689 of event type TTS); Lane 9 and 10, *PhEIN2* (Event id 1013885 of event type AE) by PCR. Abbreviations: Air, Air treatment; Eth, Ethylene treatment.

291

12.1%

893

37.1%

1224

50.8%

Phosphorylation

Ubiquitination

**Supplementary Figure S7** Venn diagram of phosphorylated proteins and ubiquitylated proteins.
